# Tensile and Flexural Behavior of Metal–Polymer Friction Stir Buttstrap Composite Panels

**DOI:** 10.3390/polym17152084

**Published:** 2025-07-30

**Authors:** Arménio N. Correia, Daniel F. O. Braga, Ricardo Baptista, Virgínia Infante

**Affiliations:** 1 Instituto Superior Técnico, Universidade de Lisboa, Av. Rovisco Pais, 1049-001 Lisboa, Portugal; 2 INEGI, Faculdade de Engenharia da Universidade do Porto, Rua Dr. Roberto Frias, 400, 4200-465 Porto, Portugal; 3 UnIRE, ISEL—Instituto Instituto Superior de Engenharia de Lisboa, Instituto Politécnico de Lisboa, Rua Conselheiro Emídio Navarro, 1959-007 Lisboa, Portugal; 4 LAETA, IDMEC, Instituto Superior Técnico, Universidade de Lisboa, Av. Rovisco Pais, 1049-001 Lisboa, Portugal

**Keywords:** friction stir joining, composite joints, tensile strength, flexural strength, defects, hardness

## Abstract

This study investigates the friction stir joining of AA6082-T6 aluminum alloy and Noryl GFN2 polymer in a buttstrap configuration, targeting the development of lightweight cylindrical-shaped structures where the polymer provides thermal, chemical, and electrical insulation, while the aluminum ensures mechanical integrity. A parametric analysis was carried out to assess the ability to produce friction stir buttstrap composite panels in a single processing step and assess the resulting tensile and flexural behavior. To that end, travel and rotating speeds ranging from 2150 to 2250 rpm, and 100 to 140 mm/min, respectively, were employed while keeping plunge depth and the tilt angle constant. A total of nine composite joints were successfully produced and subsequently subjected to both tensile and four-point bending tests. The tensile and flexural strength results ranged from 80 to 139 MPa, and 39 to 47 MPa, respectively. Moreover, the microstructural examination revealed that all joints exhibited a defect within the joining region and its size and shape had a significant effect on tensile strength, whereas the flexural strength was less affected with more uniform results. The joining region was also characterized by a decrease in hardness, particularly in the pin-affected region on the aluminum end of the joint, exhibiting a W-shaped pattern. Contrarily, on the polymeric end of the joining region, no significant change in hardness was observed.

## 1. Introduction

The transport sector is one of the key drivers of global economic growth yet, despite its significant contribution to increasing social welfare, it is also accountable for growing levels of energy consumption and greenhouse gas emissions [1,2]. The transportation sector accounts for the second-largest source of global emissions that negatively impacts the environment and induce global warming [2,3].

Seeking an ambitious balance between fostering social welfare and economic growth, while transitioning to a climate neutral transportation sector, a framework of programs that include both incentives and policies have been designed and implemented in Europe. Programs such as the EU Battery Alliance, Clean Hydrogen Partnership, or Horizon Europe, are examples of programs specially designed to promote the transition to battery-based electric vehicles (BEVs), hydrogen fuel cell vehicles (HFCVs), or both [4,5,6]. They are targeted to promote and prioritize research and innovation towards the adoption of new and disruptive technologies that enable fast and continuous change, particularly in the automotive and aerospace sectors [3]. This requires that all players involved in the transport sectors, namely, transport companies, energy suppliers, infrastructures, and others, to synergistically cooperate with each other to achieve the common goal of climate neutral mobility.

Concerning vehicle manufacturing, a growing use of a combination of metals and polymer matrix composites has been observed, exploiting the benefits offered by each of them. This structural hybridization enables the incorporation of complex design solutions that foster weight savings and multi-functional capabilities, intimately aligned with the current environmental requirements [7]. Despite the inherent potential, the widespread application of metal–polymer hybrid structures is considerably constrained by technological challenges associated with joining dissimilar materials. These challenges are mostly related to the significant differences in physico–chemical and thermo-mechanical characteristics that hinder their compatibility and, consequently, their joinability [8,9].

Mechanical fastening and adhesive bonding have been the main joining technologies employed to combine such dissimilar materials, each with their own technological constraints [10,11]. In what refers to mechanical fastening, it is typically characterized for its ease of assembly and disassembly, which benefits both maintenance and repair operations in structural components; however, it also requires added weight to balance the stress concentration around the fastening holes [12,13]. In turn, adhesive bonding offers the ability to spread the loads over a larger area, reducing stress concentrations, while acting as vibrations dampers [14]. However, adhesives are also highly susceptible to adverse environmental conditions such as service temperature and moisture [11].

In the early 1990s, a solid-state joining technology known as friction stir welding (FSW) was developed and patented by Wayne Thomas at The Welding Institute (TWI) [15,16]. In FSW, and its derivative technologies, a rotating tool is plunged into the base materials, where friction at the contact surfaces—combined with intense plastic deformation—generates heat along a desired joining path. This localized heating, together with the applied downward forging force, enables the joining of the materials without emission of fumes while reducing the overall processing energy consumption [7,17,18,19].

In recent years, FSW-derived technologies have seen growing application in the joining of dissimilar materials, with significant progress particularly in the development of similar and dissimilar polymeric joints, as well as metal–polymer composite joints [18,19,20,21]. Friction stir joining technologies offer several advantages when employed to fabricate metal–polymer hybrid structures. Compared to traditional welding, the processing temperatures in solid-state joining are typically below the melting temperature of the metallic base materials, thereby minimizing thermal degradation of polymers [22,23]. Moreover, the localized and controlled heat input results in low thermal distortion that, combined with short processing times, make this technology suitable for highly automated and scalable production. Since the binding mechanisms primarily rely on strong mechanical interlocking features at the interface, the use of specialized surface pre-treatments or filler materials is not required [24,25,26]. Consequently, the overall structural weight can be reduced, while simultaneously eliminating the need for adhesives and mechanical fasteners, along with the above-mentioned limitations, resulting in potential cost savings and enhanced manufacturing efficiency [8].

These developments have established solid-sate joining as an appealing competitor to conventional mechanical fastening and adhesive bonding, garnering interest in industries such as automotive and aerospace.

Arici and Sinmazçelýk [27] researched the impact of employing a double-pass FSW tool to join polyethylene (PE) sheets with 3 and 5 mm of thickness in overlap configuration. The joints were produced using a combination of rotational speeds ranging from 600 to 1000 rpm, travel speeds between 12.5 and 60 mm/min, as well as tilt angles spanning from 0 to 1°. The authors observed a positive effect of using double-pass with high rotational speed and tilt angle to mitigate the unwelded root defect, resulting in increased tensile and flexural strengths efficiencies around 81% of the base material strength.

Mirabzadeh et al. [28] carried out a design of experiments to assess the influence of tilt angle, shoulder to pin ratio, as well as both rotational and travel speeds on flexural strength of friction stir joined polymeric panels. The authors used polypropylene sheets with 6 mm of thickness positioned in butt joint configuration. The flexural strength was assessed by subjecting the specimens to three-point bending tests with results ranging from ~23 to ~36 MPa, equating to flexural efficiencies between ~76% and ~106% compared to base material strength, corroborating the results obtained by Arici and Sinmazçelýk [27]. The analysis of variance revealed that the rotational speed had the most influence on flexural strength while tilt angle had the least effect.

Derazkola et al. [29] investigated metal–polymer joining using friction stir lap welding to combine AA5058 aluminum alloy and PMMA polymer, both with a thickness of 4 mm. The experimental setup employed constant rotational and traverse speeds of 1600 rpm and 25 mm/min, respectively, while varying the tilt angle (0–2°) and plunge depth (0.1–0.4 mm). The overlapping configuration was characterized by the polymeric sheet being placed over the metallic one. The optimal joint, obtained with a plunge depth of 0.2 mm and 2° of tilt angle, exhibited an ultimate tensile strength (UTS) of approximately 45 MPa, equating an efficiency of 58%. The authors identified mechanical interlocking with interfacial hook formation from the aluminum plate, and chemical bonding as the main binding mechanisms. The predominant failure mode involved detachment of the polymeric material from the aluminum hooks.

Similarly, Huang et al. [25] explored the joinability of 2.5 mm thick AA6061-T6 aluminum alloy with 7 mm thick PEEK, also via friction stir joining in overlap configuration. The metallic plate was placed on top of the polymeric one. The use of a tapered-thread, triple-facet pin tool enabled the formation of aluminum hooks that anchored into the polymer, in line with the interlocking mechanisms observed by Derazkola et al. [29]. The pin-affected region was characterized by aluminum chips embedded into the polymer matrix, which overflowed onto the aluminum surface, a morphology previously reported by Correia et al. [26]. This morphology induces discontinuities in the metallic plate that adversely affect the structural integrity of the joint. The joint fabricated at 50 mm/min and 900 rpm failed by partial detachment of the aluminum plate through the hook interface, exhibiting a maximum shear strength around 20 MPa that corresponds to an efficiency of 21%, which is considerably lower than those obtained by Derazkola et al. [29].

Correia et al. [12] carried out an experimental and numerical analysis to assess the failure behavior of aluminum–polymer friction stir overlap composite joints. The joints were produced using Noryl GFN2 and AA6082-T6 with 5 and 2 mm of thickness, respectively. The fabrication process was position-controlled keeping a shoulder penetration of 0.2 mm into the aluminum plate, 1136 rpm of rotational speed, 154 mm/min of travel speed, and a tilt angle of 1.7° leaning backwards. All specimens failed by crack nucleation at the transition zone between the joining region and the polymeric base material with instantaneous propagation across the polymeric plate. Both numerical and experimental data indicated that the development of secondary bending moment acted as main catalyst of the failure mechanism since it locally increased the strain level, leading to crack nucleation at that location limiting the load bearing capacity of the joints. The bending stress component accounted for 2/3 of the total stress, whereas the remaining 1/3 was due to tensile component, limiting the load bearing capacity of the joints.

In this study, metal–polymer composite panels were fabricated using friction stir joining in buttstrap configuration. This joining configuration aims to (i) mitigate the development of secondary bending moment inherent to overlap joints typically employed to join metals and polymers, and (ii) enable the design and manufacturing of lightweight cylindrical-shaped structures where the polymer provides thermal, chemical, and electrical insulation, while the aluminum ensures mechanical integrity. To this end, a parametric analysis was conducted by varying the rotational and travel speeds to evaluate their influence on joint quality. The resulting joints were comprehensively characterized through tensile and flexural testing, assessment of both macro- and microstructural morphology, as well as hardness profiling the joining region.

## 2. Materials and Methods

In the present research work, the buttstrap joints were produced using 2 mm thick AA6082-T6 aluminum alloy and 5 mm thick Noryl GFN2 as the base materials. AA6082-T6 is a medium-strength aluminum alloy widely used in the manufacture of structural components, not only for its mechanical strength but also for its good weldability, machinability, and corrosion resistance [30]. Noryl GFN2 is a blend of poly (phenylene ether) (PPE) and high-impact polystyrene (PS) reinforced with 20 wt.% short glass fibers, and it is formed by injection molding at temperatures ranging from 299 to 327 °C. Since PPE and PS are completely miscible, their blend results in an amorphous thermoplastic with uniform microstructure and stable properties. This compatibility is essential to achieve the desirable combination of properties exhibited by the polymer, which includes very low moisture absorption, high strength, hydrolytic stability, and excellent dimensional stability [31,32]. Table 1 lists the main physical and thermo-mechanical properties of Noryl GFN2 and AA6082-T6.

**Figure 1:**
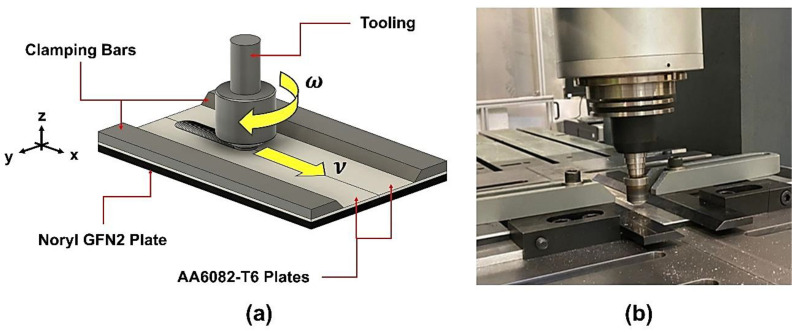
(**a**) Schematic of friction stir joining process (not to scale), and (**b**) buttstrap joining procedures setup.

The fabrication process was carried out using a custom-built FSW machine equipped with a modular tool consisting of a 0.4 mm long, 5 mm diameter threaded cylindrical pin, attached to a flat scrolled shoulder with a diameter of 16 mm. The aluminum sheets were cut into 300 × 62.5 mm plates and rectified to ensure proper surface contact, while the polymeric sheets were cut into 300 × 125 mm plates. Based on previous research over metal–polymer lap joints [11] and aluminum butt joints [34], the parametric study encompassed the production of six composite panels employing fixed tilt angle (α) and plunge depth (PD)—0° and 0.7 mm, respectively, combined with the set of parameters listed in Table 2, and following the setup displayed in Figure 1.

**Figure 2:**
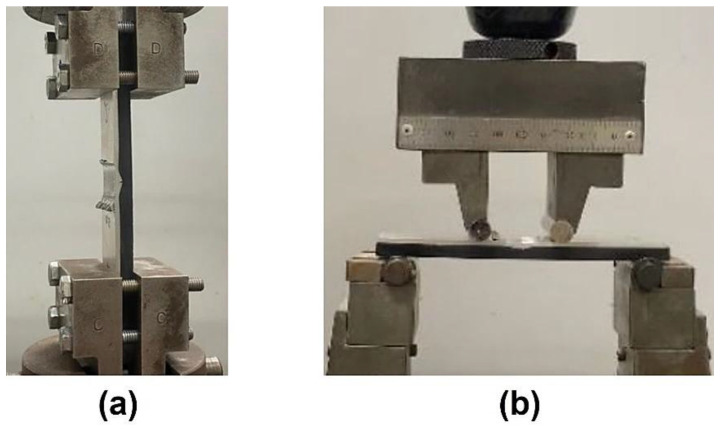
Mechanical testing setup: (**a**) tensile test, and (**b**) four-point bending test.

The composite panels were cut perpendicularly to the joining path into specimens with widths of 5 mm and 25 mm for subsequent testing and analysis. The 25 mm wide specimens were subjected to tensile and four-point bending tests performed at 5 and 2.9 mm/min, respectively. During tensile tests, both ends of the specimens were clamped across all the composite thickness and subsequently pulled. In turn, the setup of the flexural tests entailed the orientation of the polymeric end of the joint to the tension side, as this base material exhibits the lowest mechanical strength, thus ensuring a conservative estimate of the joint’s performance under load. The span lengths of the supporting and loading rollers were set to 105 and 35 mm, respectively, establishing a span ratio of 1:3. This setup induces a uniform stress distribution across the specimen, minimizing the influence of localized effects such as shear stress, allowing for more accurate measurement of the panels’ flexural behavior [35]. The tensile and flexural tests were conducted using an Instron 5566 universal testing machine (Norwood, MA, USA). The specific setup of the machine is shown in Figure 2.

The 5 mm wide specimens were cold mounted and polished to facilitate detailed microstructural analysis. Scanning electron microscopy (SEM) was employed to examine the microstructural morphology of the joining region using an analytical SEM (Hitachi S2400, Tokyo, Japan). The samples’ surfaces were coated with Au–Pd alloy to prevent the accumulation of charge during analysis, and the images were acquired in secondary electrons mode to enable high-resolution imaging for detailed characterization of the joining region, identifying critical morphological features. Additionally, microhardness profiling was performed using a Shimadzu HMV-2 microhardness tester (Kyoto, Japan) at both metallic and polymeric ends of the joints.

## 3. Results and Discussion

The visual inspection of the composite panels revealed a general high-quality surface finish, characterized by a negligible amount of flash, well-defined surface onion rings, and clean exit holes. These characteristics are typically indicative of sound joints produced by friction stir-based technologies [9,36]. Panels 1 and 2 evidenced the smoother seam surfaces, whereas Panels 5 and 6 evidenced rougher ones and the difference can be explained by the process pitch (ratio between travel and rotational speed) employed. As reported by Chinchanikar et al. [37], the roughness of seam surfaces increase with increasing process pitch, as is the present case.

**Figure 3:**
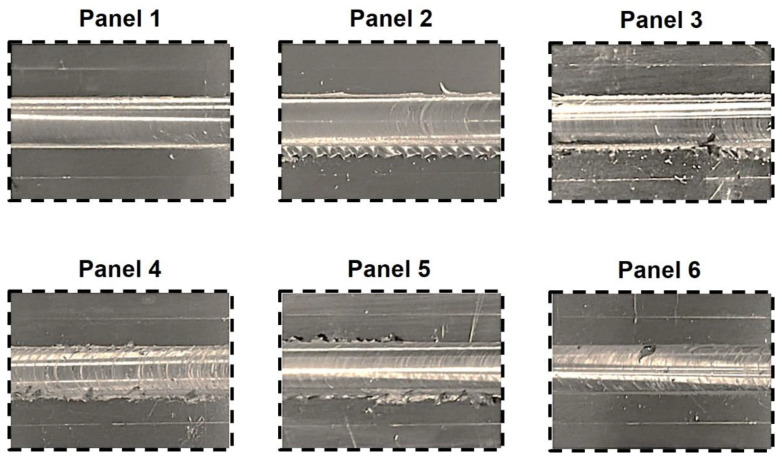
Details of the surface quality of each of the friction stir buttstrap composite panels.

Moreover, regardless of the employed process parameters, all fabricated panels exhibited a visible defect at the interface among the three plates, that will be comprehensively analyzed in following subsections. The processed region’s surface quality associated to each friction stir buttstrap composite panel produced is portrayed in Figure 3.

The friction stir buttstrap panels exhibit an asymmetric metal–polymer cross-section and, for that reason, a complex stress pattern through thickness is expected. To overcome this analysis challenge, an equivalent homogeneous cross-section can be determined using the transformed section method, converting the composite section into an equivalent one made of a single material [38]. To that end, a transformation factor (n) must be determined as per Equation (1):

(1) $$\begin{array}{c} n=\frac{E_{Aluminum}}{E_{Noryl}}=\frac{70}{6}=11.67 \end{array}$$

**Figure 4:**
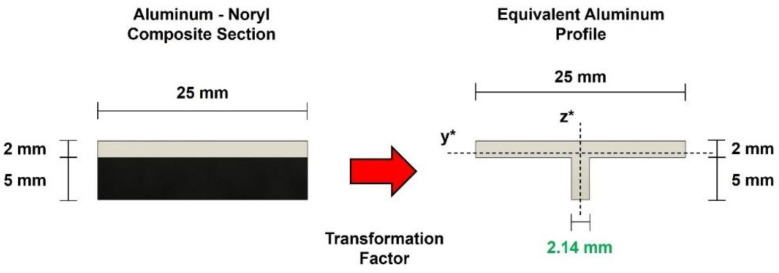
Transformed composite section geometry.

Considering the nominal thickness of each base material, and applying the transformation factor to the polymeric portion of the composite section, an equivalent homogeneous aluminum cross-section can be derived, as shown in Figure 4:

As a result, the analysis of both tensile and flexural strength will be based on the equivalent aluminum section with the properties listed in Table 3.

### 3.1. Tensile Strength

The tensile test results revealed significant variation among the composite panels. Panel P2 exhibited the highest tensile strength at 138.9 ± 9.6 MPa, while Panel P6 showed the lowest value at 80.3 ± 6.7 MPa, representing a 48% difference in tensile performance between the best and worst cases. Notably, both panels were fabricated using the same rotational speed of 2250 rpm, yet Panel P6 was processed with a travel speed 40% higher, corresponding to a significantly higher process pitch that, in turn, results in an expected lower processing temperature [22]. This suggests a detrimental effect of significantly increase the travel speed on tensile strength of buttstrap joints.

**Figure 5:**
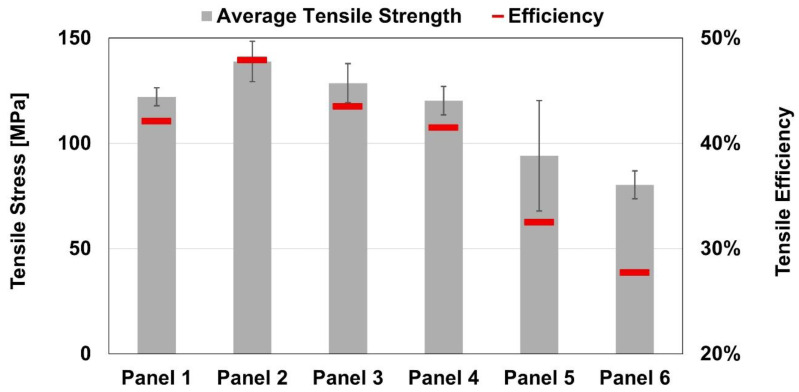
Average tensile strength and efficiency of the composite panels.

Since all failures occurred across the full thickness of the composite cross-section (as detailed later), and considering the previously calculated transformed section, the results were normalized against the tensile strength of the AA6082-T6 base material. Based on this comparison, the tensile efficiency of the joints ranged from 27.7 to 47.9%. The tensile test results are displayed in Figure 5.

### 3.2. Flexural Strength

In contrast to the tensile results, the four-point bending tests exhibited significantly less variability in flexural strength results. Panel P1 displayed the highest flexural strength of 47.2 ± 0.1 MPa, while Panel P3 showed the lowest value of 41.9 ± 4.7 MPa, representing an approximate 11% difference between the best and worst performing panels. When normalized in terms of flexural efficiency, the values ranged from 52.4 to 59.0%, further highlighting the consistency and reliability of the flexural performance across specimens. In this analysis, the tensile strength of Noryl GFN2 was used as the reference, since solely the polymeric base material fractured, whilst the metallic end of the panels evidenced significant amount of plastic strain instead.

**Figure 6:**
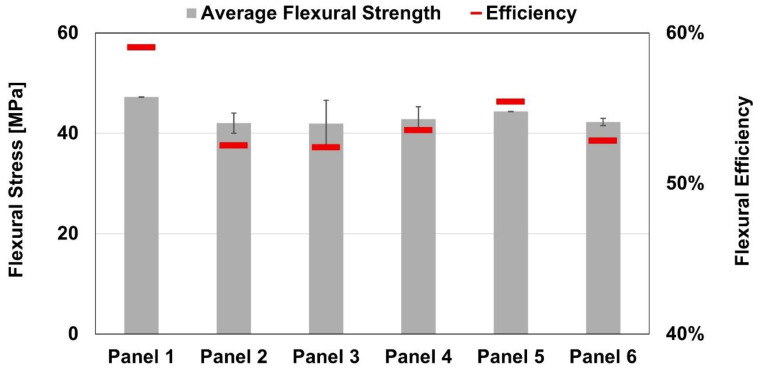
Average flexural strength and efficiency of the composite panels.

Despite the relatively consistent results, an apparent negative correlation between travel speed and flexural strength was observed. Both panels were processed at the same rotational speed of 2150 rpm, yet Panel P3 was joined with a 20% higher travel speed, suggesting that, similarly to the tensile results, the increase in travel speed may adversely affect flexural properties due to lower heat input. Further details on four-point bending tests results can be observed in Figure 6.

### 3.3. Microstructure and Fracture Surfaces

To investigate the morphological features potentially underlying the discrepancies observed, particularly the results obtained in the tensile tests, a comprehensive analysis of microstructural characteristics of the joint cross-sections was conducted. In order to thoroughly characterize the joining interfaces and correlate them with mechanical tests, Panels P2 and P6 were selected for SEM analysis. This selection was based on their notable difference in tensile strength, representing the highest and lowest tensile performance, respectively.

**Figure 7:**
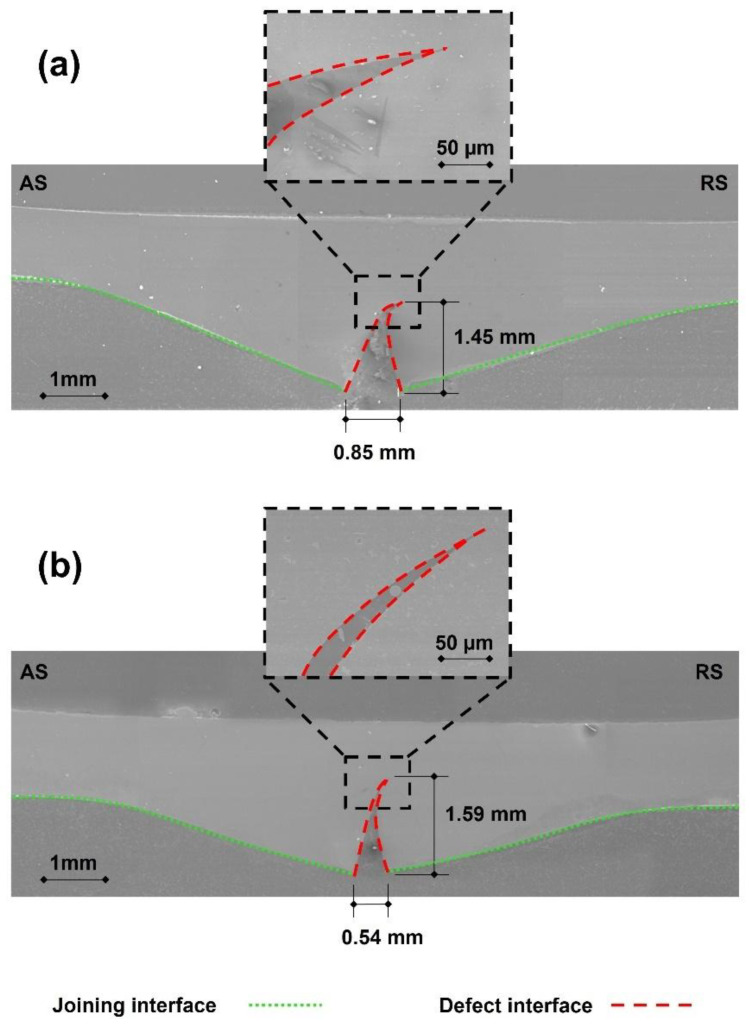
SEM images of composite panels and details of the joining defects in panels (**a**) P2 and (**b**) P6. AS and RS stand for advancing and retreating side, respectively.

The morphology of the joining region of both panels was found to be very similar, with the aluminum plates being clinched into the polymeric one. The interface between the metallic and polymeric base materials showed a prominent double concavity shape, a geometrical feature that induces macro-mechanical interlocking [11]. This joining region’s morphology evidence that the aluminum plates were welded and extruded downwards into the polymeric molten pool due to the vertical force exerted by the tool. Despite the fact that this morphology enables the development of the main binding mechanism in metal–polymer friction stir joints, as previously reported by Correia et al. [22,26], a notable gap filled with polymeric inflow was found at the interface between the three base material plates, rather than a smooth transition between concavities. This defect shares a combination of traces that are typically found in butt and overlap joints—the unwelded root defect [27,39,40], and the hook shaped defect [41,42], respectively. Further details on the morphology of the joining regions are depicted in Figure 7.

The defect in panel P6 was found to be 10% longer and 36% narrower compared to the defect in panel P2. As a result, not only the effective aluminum thickness is smaller in panel P6, but also a higher stress concentration is expected given the sharpness of the defect. Furthermore, the asymmetric gradient of mechanical properties along the cross-section of the composite panels will promote a local shifting in the position of the neutral axis, inducing the development of secondary bending moment. As a result, the local stress will be further increased, intensifying the sensitivity of the joints under tensile loading to the presence of such defects, as previously reported by Correia et al. [12,22].

These results corroborate the tensile strength difference among the composite panels, suggesting that increased volume of upward flow of the molten polymer deteriorates not only the metallurgical weldability between aluminum plates but also further increases the local stress. This way, the polymeric inflow will, ultimately, compromise the structural integrity of the joints and their load bearing capacity. Contrarily, in what refers to four-point bending tests, the observed low variability in the results suggests that the interfacial defects did not have a significant effect on the flexural performance of the composite panels.

**Figure 8:**
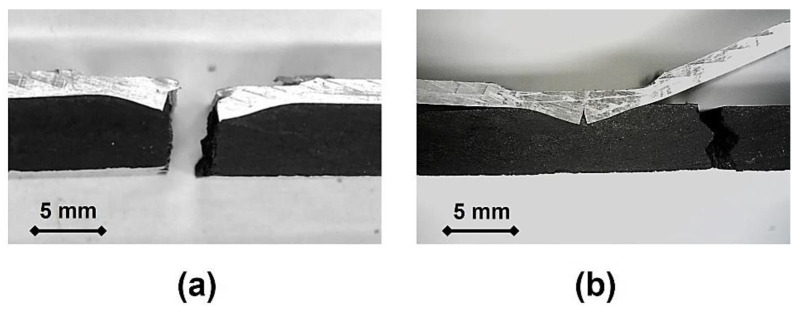
Fracture surfaces of panel P2 submitted to (**a**) tensile and (**b**) flexural tests.

By examining the fracture surfaces of the tensile-tested specimens, it was observed that these defects consistently served as crack initiation sites due to increased localized stress, as previously suggested. Once initiated, the cracks rapidly propagated across the cross-section of the composite plates, leading to complete failure, as shown in Figure 8a. The influence of this defect on structural integrity of the joint under tensile loadings suggests that an alternative geometry of the tool, as well as an optimized set of processing parameters, should be considered to decrease or even mitigate the polymeric inflow into the metallic side of the joint.

In turn, the fracture surfaces of the bending tested specimens—shown in Figure 8b—were found to solely affect the polymeric base material, whereas the metallic end evidenced a significant amount of plastic strain. The fracture nucleated and propagated under the line of action of the loading roller, away from the defect in the joining region, further supporting its marginal effect on flexural performance.

### 3.4. Hardness Profile

The panels selected for SEM analysis were also subjected to hardness testing and profiling. Considering the metal–polymer nature of the composite joints, measurements were performed on both the metallic and polymeric regions. The baseline hardness values of the base materials were previously determined, with AA6082-T6 exhibiting a hardness of 102 HV_1_ and Noryl GFN2 recording 18 HV_0.2_, respectively [11].

**Figure 9:**
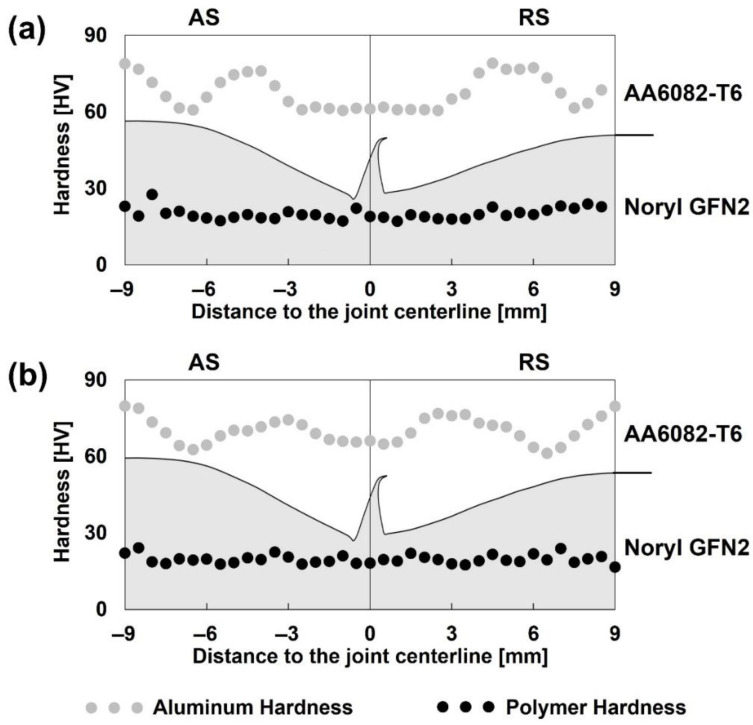
Hardness profiles on composite panels: (**a**) Panel 2, and (**b**) Panel 6.

The hardness profiles presented in Figure 9 demonstrate that changes in processing parameters had no meaningful influence on the overall hardness distribution. On the aluminum side, the profile exhibits a distinct W-shaped pattern, a behavior associated with heat-treatable aluminum alloys [43,44]. In contrast, the polymeric portion of the joint exhibited a consistent hardness close to the reference value (18 HV_0.2_), indicating that the processing conditions did not significantly jeopardize the polymer’s mechanical properties. The thermal stability observed in the polymeric base material indicates that the heat input during processing was sufficiently localized. This, combined with the material’s inherent thermal insulating properties, effectively prevented a broad thermal degradation in this base material [11].

On the one hand, the localized softening observed on the aluminum side of the joint, combined with the detrimental influence of defects within the joining region, are likely the primary factors contributing to the tensile performance variation, which ranged from 27.7% to 47.9%. On the other hand, given that the defects exhibited a marginal impact on the flexural strength, the observed reduction in hardness appears to be the main factor accountable for the flexural efficiency values, which varied between 52.4% and 59.0%.

## 4. Conclusions

In this experimental research, the tensile and flexural behavior of friction stir buttstrap-joined AA6082-T6 and Noryl GFN2 were assessed. To that end, a parametric study was carried by combining different rotational and travel speeds, with the following results:

- The tensile strength evidenced scattered results from 80.3 ± 6.7 MPa in panel P6 (2250 rpm and 140 mm/min) up to 138.9 ± 9.6 MPa in panel P2 (2250 rpm and 100 mm/min);
- The tensile strength was significantly hindered by two critical factors: (i) the presence of a defect within the joining regions, and (ii) the loss of hardness in the joining region;
- The tensile efficiency ranged from 27.7% to 47.9% with tensile strength of AA6082-T6 as reference value;
- The lowest flexural strength was observed in panel P3 (2150 rpm and 120 mm/min), with 41.9 ± 4.7 MPa, while the highest was found in panel P1 (2150 rpm and 100 mm/min), with 47.2 ± 0.1 MPa, evidencing stable results compared with the tensile ones;
- The flexural efficiency varied between 52.4% and 59.0%, with marginal contribution from the defect while the hardness loss played a major role in this domain;
- The travel speed during the joining process appears to have had a negative effect on mechanical performance.

These results substantiate the attractiveness of employing a friction stir-based technology for production of metal–polymer composite panels. These metal–polymer composite panels are particularly suitable to be incorporated in cylindrical-shaped structures where the polymeric material provides thermal, chemical, and electrical insulation, while the metallic one ensures the overall mechanical integrity.

Given the early stage of development, further research is required to ensure the mitigation of the presence of defects within the joining region to increase the overall performance of such composite panels.

## Figures and Tables

**Table 1:** Main thermo-mechanical and physical properties of Noryl [33] and AA6082-T6 [30].

Base Materials	ρ (g/cm^3^)	E (GPa)	$\sigma_{UTS}$ (MPa)	$\varepsilon_{f}$ (%)	T_melt_ (°C)	K (W/(m °C))
Noryl GFN2	1.25	6	80	2.5	280	0.26
AA6082-T6	2.70	70	>290	>7	582	180

**Table 2:** Variable and constant processing parameters associated to each composite panel.

Parameter	Panel 1	Panel 2	Panel 3	Panel 4	Panel 5	Panel 6
ω (rpm)	2150	2250	2150	2250	2150	2250
v (mm/min)	100	100	120	120	140	140

ω: Rotating Speed; v: Travel Speed.

**Table 3:** Equivalent aluminum section properties.

E (GPa)	$A^{*}$ (mm^2^)	$z_{c}^{*}$ (mm)	$I_{yy}^{*}$ (mm^4^)
70	60.7	5.38	146.9

*E*: Young Modulus; A*: Equivalent area; $z_{c}^{*}$: Equivalent vertical position of neutral axis; $I_{yy}^{*}$: Equivalent second moment of area.

## Data Availability

The original contributions presented in this study are included in the article. Further inquiries can be directed to the corresponding author.
